# Newly developed short-length exposed-tip intraductal radiofrequency ablation probe for intra-ampullary adenocarcinoma of the ampulla of Vater

**DOI:** 10.1055/a-2739-2482

**Published:** 2025-11-21

**Authors:** Mitsuru Okuno, Fumiya Kataoka, Tsuyoshi Mukai, Hiroshi Araki, Eiichi Tomita, Hisataka Moriwaki, Masahito Shimizu

**Affiliations:** 1 73505 Department of Gastroenterology, Matsunami General Hospital, Gifu, Japan; 2 476117 First Department of Internal Medicine, Gifu University Hospital, Gifu, Japan

Carcinomas of the ampulla of Vater can cause biliary stricture and jaundice. Intraductal radiofrequency ablation (RFA) has recently been used to treat biliary neoplasms 1 , and a short-length exposed tip RFA probe (11-mm; ELRA, STARmed, Goyang, Korea) has been developed 2 .

A 92-year-old male patient presented to our hospital with fever. Computed tomography and endoscopic ultrasound revealed a dilated common bile duct (CBD), dilated pancreatic duct, and a 6 mm mass in the main papilla. As cholangitis due to tumor obstruction was considered, endoscopic retrograde cholangiopancreatography was performed. Endoscopy revealed a bulge in a fold of the papilla without a tumor on the mucosal surface. Cholangiography revealed a 10 mm distal CBD stricture, which was biopsied, and a 7 Fr plastic stent was placed in the CBD ( Fig. 1 ). The biopsy detected adenocarcinoma, and intra-ampullary adenocarcinoma of the ampulla of Vater was diagnosed. Due to the location of the mass and the patient’s age, endoscopic papillectomy or surgical pancreaticoduodenectomy was considered difficult. Thus, we planned intraductal RFA for the malignant distal biliary obstruction (MDBO). After the guidewires were placed in the CBD and pancreatic duct, a short-length, exposed-tip ELRA was inserted into the MDBO. After RFA, the main papilla was cauterized. A 5Fr plastic stent was placed in the pancreatic duct and a 10 mm diameter metallic stent was placed in the CBD. Three months later, both stents were removed and a biopsy of the papilla showed no malignancy. No tumor stricture recurrence was observed after stent removal ( Video 1 and Fig. 2 ).

**Fig. 1:**
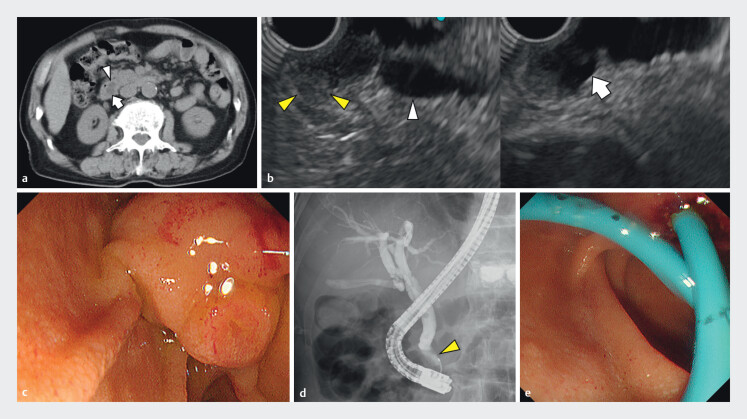
**a** Computed tomography shows the dilated common bile duct (CBD; arrow) and the pancreatic duct (arrowhead). **b** Endoscopic ultrasonography also shows the dilated common bile duct (CBD; arrow) and the pancreatic duct (arrowhead). Endoscopic ultrasonography shows a 6 mm mass in the main papilla (yellow arrowhead). **c** Endoscopy image showing a bulge in a papillary fold. No tumor lesions were observed on the mucosal surface of the main papilla. **d** Cholangiography shows a 10 mm distal CBD stricture (yellow arrowhead). ( **e** ) After obtaining tissue samples from the CBD obstruction, a 7 Fr plastic stent is placed in CBD. CBD, common bile duct; RFA: radiofrequency ablation.

Successful and safe RFA for intra-ampullary adenocarcinoma of the ampulla of Vater with a short distal CBD obstruction was achieved using a newly developed short-length exposed-tip intraductal RFA probe. CBD, common bile duct; RFA: radiofrequency ablation.Video 1

**Fig. 2:**
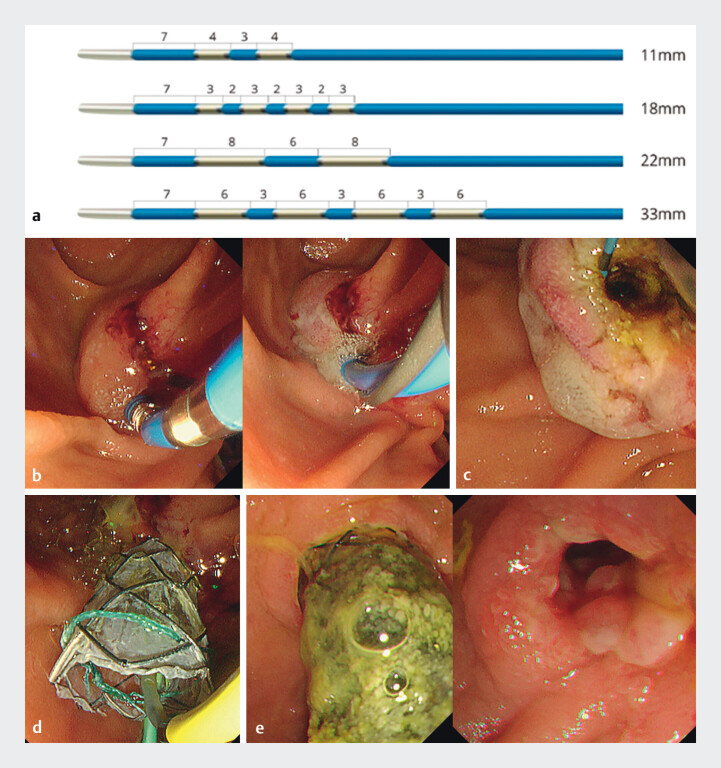
**a** A newly released intraductal radiofrequency ablation (RFA) probe (ELRA; STARmed, Goyang, Korea) has four types of exposed tip length. For this case, we used the shortest (11 mm) exposed tip length probe. **b** RFA achieved cauterization of the main papilla. **c** Endoscopy shows the outflow of bile after RFA. **d** To prevent pancreatobiliary obstruction after the RFA, a 5 Fr plastic stent was placed into the pancreatic duct, and a 10 mm diameter metallic stent was placed into the CBD. **e** Three months later, the dilated CBD without tumor obstruction is observed after the metallic stent removal. Endoscopy and the biopsy sample from the main papilla shows no malignancy in the main papilla. CBD, common bile duct; RFA, radiofrequency ablation.

Although treatment of intra-ampullary adenocarcinoma of the ampulla of Vater requires pancreaticoduodenectomy, surgical treatment was not feasible in this patient because of his age. For such patients with short MDBO, the newly developed RFA probe enabled the setting of an appropriate margin to avoid overablation, making it a useful treatment device.

Endoscopy_UCTN_Code_TTT_1AR_2AF

## Notes

Endoscopy E-Videos https://eref.thieme.de/e-videos
*E-Videos* is an open access online section of the journal *Endoscopy* , reporting on interesting cases and new techniques in gastroenterological endoscopy. All papers include a high-quality video and are published with a Creative Commons CC-BY license. Endoscopy E-Videos qualify for HINARI discounts and waivers and eligibility is automatically checked during the submission process. We grant 100% waivers to articles whose corresponding authors are based in Group A countries and 50% waivers to those who are based in Group B countries as classified by Research4Life (see: https://www.research4life.org/access/eligibility/ ). This section has its own submission website at https://mc.manuscriptcentral.com/e-videos .
